# The regulatory mechanisms and treatment of HDAC6 in immune dysregulation diseases

**DOI:** 10.3389/fimmu.2025.1653588

**Published:** 2025-09-22

**Authors:** Yanyang Liang, Ying Wang, Jianxiao Xing, Junqin Li, Kaiming Zhang

**Affiliations:** ^1^ Shanxi Key Laboratory of Stem Cells for Immunological Dermatosis, Institute of Dermatology, The Ninth Clinical Medical School of Shanxi Medical University, Taiyuan Central Hospital, Taiyuan, China; ^2^ State Key Breeding Laboratory of Stem Cells for Immunological Dermatosis, Institute of Dermatology, The Ninth Clinical Medical School of Shanxi Medical University, Taiyuan Central Hospital, Taiyuan, China

**Keywords:** HDAC6, cancer immunotherapy, neuroinflammation, autoimmune diseases, immune regulation

## Abstract

Histone deacetylase 6 (HDAC6) is a class IIb histone deacetylase that contains two catalytic domains and a zinc finger ubiquitin binding domain (ZnF-UBP). The deacetylation function of HDAC6 has been extensively studied with well-characterized substrates such as α-tubulin and Hsp90. Apart from its deacetylase activity, HDAC6 ZnF-UBP binds to unanchored ubiquitin of specific sequences and serves as a carrier for transport of aggregated proteins. subsequently, aggresomes is degraded by the autophagy-lysosome pathway. Additionally, Cells can utilize this HDAC6-dependent microtubule transport to assemble and activate inflammasomes, which play a critical role in immune regulation. HDAC6 displays a unique structure and cellular localization as well as diverse substrates, and exhibits a wider range of biological functions than other HDAC isoforms. HDAC6 has been intimately linked to a spectrum of diseases, including rheumatoid arthritis, systemic lupus erythematosus, psoriasis, neuritis, and the cancer immune microenvironment. This review systematically synthesizes the current research advancements of HDAC6, focusing on three key dimensions: the mechanism of action of HDAC6, therapeutic advancements, and translational prospects in clinical applications.

## 1 Introduction

Histone deacetylases (HDACs) are well-known as crucial epigenetic regulators. Their main role is to remove acetyl groups from lysine residues in histones and non-histone proteins. By doing so, they maintain cellular homeostasis. In cancer cells, overexpression of HDACs increases deacetylation, suppressing certain tumor suppressor genes and promoting cancer development and progression (1). In addition, HDAC enzymes also catalyze the removal of various acyl groups, such as succinyl, formyl, crotonyl, and myristoyl groups (2, 3). HDACs consist of 18 isoforms classified into two superfamilies: zinc-dependent and NAD^+^-dependent enzymes. The zinc-dependent superfamily includes class I (HDAC1, 2, 3, 8), class IIa (HDAC4, 5, 7, 9), class IIb (HDAC6, 10), and class IV (HDAC11). The NAD^+^-dependent superfamily, known as sirtuins, forms class III (Sirt1-7) (4). Histone deacetylase 6 (HDAC6) is a class IIb deacetylase, exhibits unique characteristics compared to other isoforms (5). The human HDAC6 protein comprises 1215 amino acids and includes two distinct catalytic domains (CD1 and CD2) and a zinc finger domain (ZnF-UBP domain) for ubiquitin binding (6). The catalytic domains, each approximately 360 amino acids long, rely on zinc for deacetylation. CD2 of HDAC6 from Homo sapiens and Danio rerio displays a wide range of substrate specificity compared to CD1, which is primarily due to the mouth of the CD1 active site being constricted by K330 (the similar position in CD2 was occupied by L712), thus leading to the narrow catalytic specificity of HDAC6 CD1 for peptide substrates containing C-terminal acetyl lysine residues (7). To verify the substrate specificity of CD1, researchers have synthesized a series of unnatural peptide substrates with C-terminal acetylation modifications using solid-phase peptide synthesis technology. For instance, the C-terminal lysine of natural peptide substrates was replaced with 2-amino-8-oxodecanoic acid (AODA). These unnatural peptide substrates not only serve as critical tools for elucidating the catalytic mechanism of CD1 but also lay a foundation for the development of selective HDAC6 inhibitors (8).

**Figure 1:**
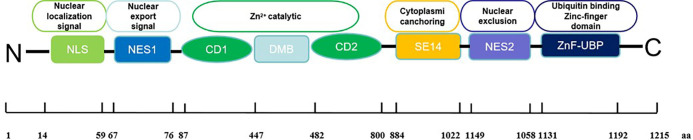
Structure of HDAC6.

Moreover, CD1 requires assistance from CD2 for its catalytic function as it cannot deacetylate independently (9). Interestingly, studies have shown that the CD1 domain can ubiquitinate unacetylated MSH2 *in vitro*, suggesting a potential E3 ligase role for HDAC6 (10). Unique to the human HDAC6 protein are the specific eight consecutive Ser-Glu-containing tetradecapeptide (SE14) repeats between the second catalytic deacetylase domain and the C-terminal ZnF-UBP, which enables its cytosolic retention (10) (**Figure 1**).

In addition to histone deacetylation, HDAC6 interacts with various non-histone substrates, including transcription factors, structural proteins, and inflammatory mediators such as α-tubulin, cortactin, HSP90, Foxp3, among others (5). Specifically, HDAC6 deacetylates α-tubulin at Lys40, a critical component of the eukaryotic cytoskeleton (11). This post-translational modification of α-tubulin is closely associated with cell division, proliferation, and migration (12). Furthermore, HDAC6 influences microtubule stability and function by acting on cortactin, a microfilament actin-binding protein, thereby promoting F-actin-dependent cell movement (13). Through deacetylation of transcription factor Foxp3, HDAC6 regulates the suppressive functions of regulatory T cells (Tregs) (14). Additionally, HDAC6 recruits and activates STAT3, modulating programmed death receptor ligand-1 (PD-L1) (15).

HDAC6 not only exerts the biological activity of deacetylating histones and non-histones, but also relies on its own zinc finger structure to clear misfolded proteins. HDAC6 exerts its function in clearing misfolded proteins by virtue of its zinc finger ubiquitin-binding domain (ZnF-UBP), and this process is primarily mediated through two cellular mechanisms: the proteasomal pathway and the aggrephagy pathway. Through the synergistic action of these two pathways, HDAC6 plays a pivotal role in maintaining intracellular protein homeostasis. On one hand, HDAC6, in collaboration with its co-factor p97/VCP (16), mediates the delivery of ubiquitinated misfolded proteins to the proteasome for degradation. On the other hand, when misfolded proteins accumulate to levels exceeding the proteasome’s processing capacity, HDAC6 facilitates their transport along microtubules to aggresomes, where subsequent degradation is executed by lysosomes. First of all, the activation of the clearance system needs protein aggresome formation mediated by HDAC6. ZnF-UBP domain of HDAC6 interacts with polyubiquitinated protein aggregates specifically via the C-terminal of the unanchored K63-linked polyubiquitin chain which is generated by deubiquitinase ataxin-3 cleavage (17, 18). Through the DMB motif, HDAC6 directly binds to dynein motors that power the transport of diverse cargo towards the microtubule minus end. Polyubiquitin-flagged protein aggregates are recruited to the microtubule-organizing center (MTOC) close to the nucleus and aggregate to form a so-called cellular aggresome. Subsequently, autophagosome encapsulates protein aggregates through the ubiquitin-recognizing receptor P62, NBR1, ALFY, etc., and completes degradation by lysosome fusion, a process called aggrephagy (5, 19).

Hsp90, a molecular chaperone that is a critical modulator of cell signaling, is one of the deacetylase substrates of HDAC6. HDAC6 also regulates Hsp90 through the ZnF-UBP domain (20). Inhibition of HDAC6 elevates HSP90 acetylation, acetylation of HSP90 reduces its chaperone activity leading to the reduction of the interaction with client proteins that are therefore ubiquitinated and degraded by proteasome (21). However, the specific mechanism by which client proteins are transported to the proteasome remains unclear. Previous studies have confirmed that HSP90 interacts directly with the 20S proteasome (22) and facilitates the transport of client proteins into the 20S proteasome (23). Based on this evidence, we hypothesize that acetylation modification may drive the functional switch of HSP90—from acting as a “chaperone protein” to a “proteasomal substrate delivery platform”—thereby promoting the degradation of client proteins. Nevertheless, the molecular mechanism underlying this functional switch still requires further investigation. For instance, in neurodegenerative disease models, HDAC6 inhibitors mitigate proteasome dysfunction induced by abnormal protein aggregation through the enhancement of HSP90 acetylation (24).

Research has linked HDAC6 to various human diseases, including tumors, inflammation, and neurodegeneration. Recent studies highlight its close association with inflammatory processes, particularly its role in assembling and activating NLRP3 inflammasomes. Hao Wu et al. have shown that dynein, along with its adapter HDAC6, transports the NLRP3 inflammasome to the microtubule organizing center (MTOC) for assembly and activation, thus triggering inflammatory responses. Additionally, studies have revealed that HDAC6 influences the transcription of inflammatory proteins and cytokines such as NLRP3, IL-1Β, and IL-6 (25). These findings provide a foundation for targeting HDAC6 in immunotherapy.

## 2 Regulation of immune cells

### 2.1 Macrophages

**Figure 2:**
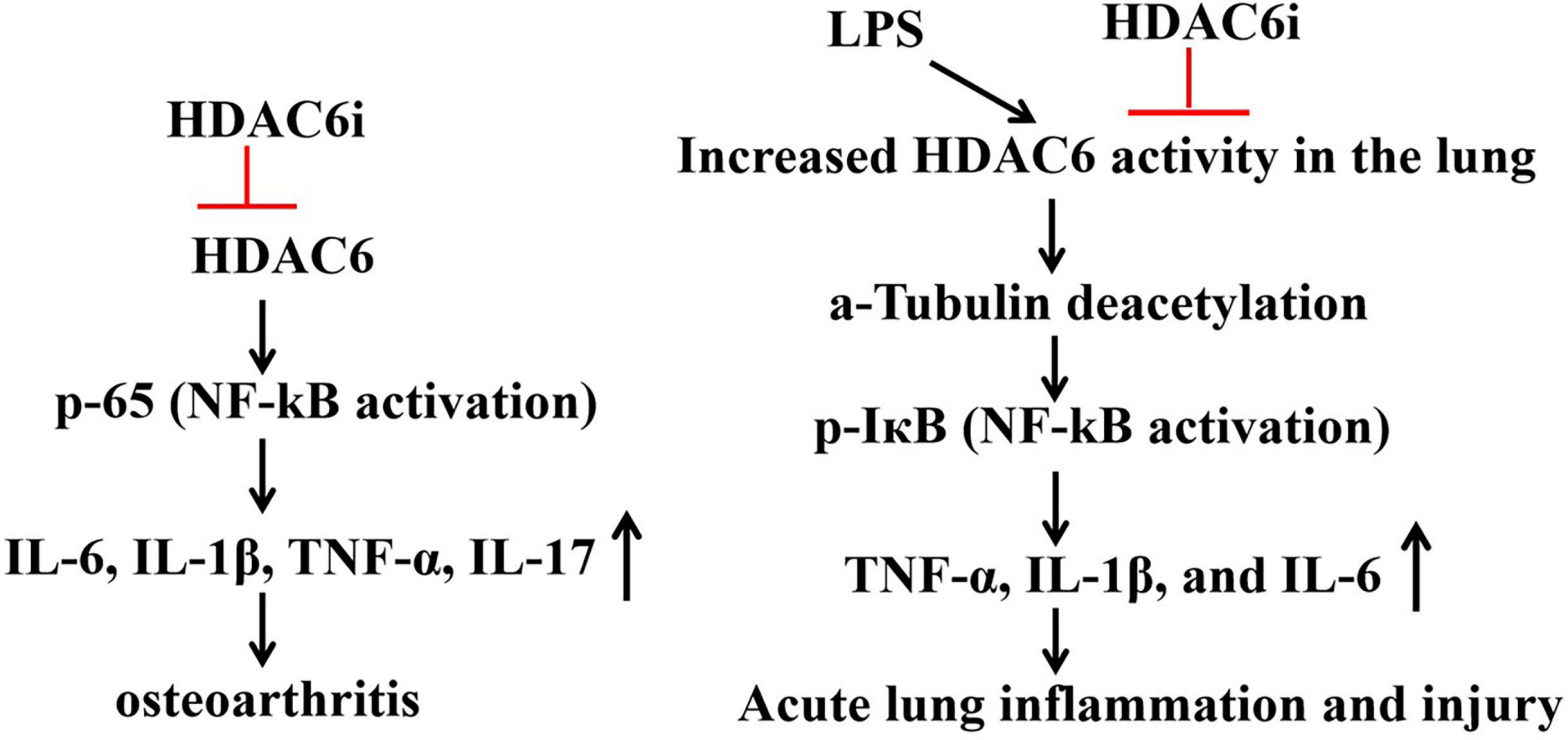
Role of HDAC6 inhibitors in IL-1B-induced osteoarthritis models and LPS-induced acute lung injury (ALI).

A recent study has demonstrated that HDAC6 inhibitors specifically suppress M2 polarization of murine macrophages with partial regulatory effects on M1 polarization. The researchers induced pro-inflammatory M1 phenotype in murine RAW264.7 cells using lipopolysaccharide (LPS) combined with interferon-γ (IFN-γ), while interleukin-4 (IL-4) plus IL-13 were used to drive anti-inflammatory M2 polarization in BMA31A7 cells. Acetylated α-tubulin levels were measured to evaluate HDAC6 inhibition by AVS100, with inducible nitric oxide synthase (iNOS) and arginase 1 (Arg1) serving as specific markers for M1/M2 polarization. As previously reported, AVS100 treatment significantly increased acetylated α-tubulin expression in both models. Notably, AVS100 potently suppressed Arg1 upregulation under M2-polarizing conditions without affecting iNOS induction in M1-polarized cells, indicating specific inhibition of M2 but not M1 polarization (26). (**Figure 2**) These findings were recapitulated in primary cultures of bone marrow-derived macrophages (BMDMs). Additionally, studies in Hdac6−/− macrophages revealed that the ubiquitin-binding domain of HDAC6 is critical for NLRP3 inflammasome activation (27). In the atherosclerosis model, the HDAC6 inhibitor Tubastatin A reduces macrophage foam cell formation and inhibits plaque progression (28).

### 2.2 Treg cells

Treg cells are a distinct subset of CD4 +T cells that prevents abnormal or excessive immune responses and development of autoimmune disorders. However, because they also suppress other effector T cells, depleting Tregs can be clinically beneficial in some cancer models. Foxp3 is a key transcription factor that is essential for differentiation and inhibitory function of Treg cells. Among all CD4 +T cell subsets examined, HDAC6 mRNA and protein levels were highest in Treg cells (29). Recent studies showed pharmacological inhibition of HDAC6 (Tubastatin A: TSA) impairs murine induced regulatory T cell(iTreg)function by downregulating Foxp3 expression (29). In melanoma, HDAC6 inhibitors induce the differentiation of Treg cells and M2 macrophages into cytotoxic T cells and M1 macrophages, thus making the tumor microenvironment immunocompetent. However, the regulatory role of HDAC6 in Treg cells varies under different environments. In rheumatoid arthritis (RA), HDAC6 inhibitors enhance the suppressive function of Treg cells (30). In systemic lupus erythematosus (SLE), HDAC6 inhibition has the capacity to reverse these trends by elevating serum levels of TGF-Β to induce a Treg phenotype (31), while reducing TGF-Β in the glomeruli of the kidneys, which potentially leads to fibrosis.

### 2.3 B cell, T cell

Development of chronic lymphocytic leukemia (CLL) is associated with severe immune dysfunction. T-cell exhaustion, immune checkpoint upregulation, and increase of regulatory T cells contribute to an immunosuppressive tumor microenvironment. Previous studies verified that HDAC6 inhibition exerts beneficial immunomodulatory effects on CLL B cells and alleviates CLL-induced immunosuppression of CLL T cells. In the Em-TCL1 adoptive transfer murine model, genetic silencing or inhibition of HDAC6 reduced surface expression of programmed death-ligand 1 (PD-L1) on CLL B cells and lowered interleukin-10 (IL-10) levels (32).

Studies showed that ACY-738 decreased several characteristics of SLE in NZB/W mice by dictating B cell development in the bone marrow. They found that there was a decrease in the percentage of cells in early B cell developmental stages and an increase in the number of cells in late B cell BM developmental stages during disease in NZB/W mice. ACY-738 treatment increased the percentage of B cells in early developmental stages, while decreasing the percentage of cells in late preB cell fraction F (31).

Forkhead box protein O1 (FoxO1), a member of the Fox family, shuttles back and forth between the nucleus and cytoplasm. HDAC6 can promote the nuclear translocation and stability of FoxO1 by deacetylating FoxO1 on K424, concomitantly inhibiting the activity of transcription factor retinoic acid-related orphan receptor gamma (RoRγt), thereby blocking the differentiation of T helper cell 17 (Th17) cells and T cell mediated antitumor immune response (**Figure 2**).

### 2.4 Regulate inflammatory factors

HDAC6 binds to NLRP3 through the ZnF-UBP domain, promoting its transport the from TGN to the microtubule tissue center (MTOC), forming a functional NLRP3 inflammasome complex, activating caspase-1 and releasing IL-1Β and IL-18. Researchers found that in Hdac6−/− mice macrophages, the secretion of IL-1Β induced by LPS/ATP decreased by 50%, indicating that HDAC6 is a key factor for the activation of NLRP3 (27). ACY-1215 (Ricolinostat) is a HDAC6 inhibitor and holds potential as an anti-inflammatory agent (33). In IL-1Β-induced osteoarthritis models, Rocilinostat prevents the phosphorylation and subsequent translocation of p65 to the cell nucleus, thereby inhibiting the expression of inflammatory cytokines (IL-6, IL-1Β, TNF-α, IL-17) through the suppression of the NF-ΚB pathway (34). The HDAC6 inhibitor CAY10603 mitigates LPS-induced pulmonary microtubular deacetylation, leading to a reduction in the production of inflammatory cytokines TNF-α, IL-1Β, and IL-6, as well as a decrease in white cell infiltration. HDAC6 inhibitors impede the activation of NF-ΚB by inhibiting IΚB phosphorylation in LPS-induced acute lung injury, indicating that HDAC6 selectively targets inflammatory signaling pathways and alleviates LPS-induced acute lung injury (35) (**Figure 2**).

## 3 HDAC6 as a therapeutic target for immune dysregulation-related diseases

### 3.1 Autoimmune diseases

#### 3.1.1 Rheumatoid arthritis

Rheumatoid arthritis (RA), a chronic autoimmune condition, primarily affects peripheral joints, leading to deformities (36). Pathologically, it is characterized by continuous synovitis, vascular changes, and bone erosion. Abnormal proliferation of synovial tissue plays a critical role in RA development (37). RA fibroblast-like synoviocytes (RA-FLS), specialized mesenchymal cells in the synovium of affected joints, are key contributors to RA progression (38). These cells secrete various pathogenic mediators, such as cytokines (tumor necrosis factor alpha (TNF-a) and interleukin-6 (IL-6), which promote the migration and differentiation of other cells in the synovial membrane, accelerating RA. Therefore, RA-FLS cells were used as a model for rheumatoid arthritis. CAY10603, a specific inhibitor of HDAC6, potently suppressed the proliferation of RA-FLS cells at 10 μM. Nevertheless, despite its inherent specificity for HDAC6, a 10 μM concentration may surpass the threshold where such specificity is preserved. As a result, the biological effects observed at this concentration could imply that it also exerts inhibitory activity against other members of the HDAC family. Furthermore, ELISA analysis of supernatants derived from RA-FLS cells demonstrated that CAY10603 diminished the secretion of the proinflammatory cytokines TNF-α and IL-6 (39). However, in another related study, HDAC3 inhibitors effectively suppressed the expression of IL-1Β, whereas neither inhibition nor silencing of HDAC6 exerted a corresponding effect in RA-FLS cells. Therefore, extensive experiments are still required to explore the regulatory role of HDAC6 inhibitors in inflammation within the RA-FLS cells (40) (**Table 1**).

**Table 1:** HDAC6 inhibitors used as a treatment for rheumatoid arthritis (RA).

HDAC6 inhibitor(s)	Model	Outcome	Pathogenic mechanism(s)	Reference
CAY10603	RA-FLS cell	CAY10603 effectively attenuated the proliferative effect of RA-FLS cell	CAY10603 downregulated the secretion of TNF-α and IL-6 inflammatory factors in RA-FLS cell.	(39)
CKD-L	CIA mice	CKD-L significantly decreased both the arthritis score and the histological score	CKD-L promotes the suppressive function of Treg cells by increasing expression of Foxp3.	(14, 30)
CKD-506	FLS and AIA rats	Oral CKD-506 improved clinical arthritis	CKD- 506 inhibited NF-ΚB activation and production of MMP-1, MMP-3, IL-6, and IL-8.	(44, 45)
M-134	AIA CIA mice	M-134 improved the clinical arthritis score	M-134 downregulated various cytokines, such as interleukin IL-1Β, IL-17, and TNF-α.	(46)
M808	RA-FLS cell	In the AIA arthritis model, M808 improved the clinical arthritis score in a dose dependent manner.	M808 suppressed the production of MMP-1, MMP-3, IL-6, CCL2, CXCL8, and CXCL10 in RA-FLS cell.	(47)

CKD-L is a novel selective HDAC6 inhibitor. Studies have demonstrated that CKD-L can significantly reduce arthritis scores and histological scores, thereby inhibiting the progression of collagen-induced arthritis (CIA) (41, 42). Additionally, CKD-L enhances the suppressive function of regulatory T (Treg) cells, which is likely attributed to its ability to increase the acetylation levels of histones and Foxp3. This, in turn, leads to an upregulated expression of Foxp3 and promotes the generation and suppressive function of Treg cells (14, 30) (**Table 1**).

A study examined the anti-arthritic effects of CKD-506, a novel HDAC6 inhibitor (43), both *in vitro* and in a murine arthritis model, as a potential treatment for rheumatoid arthritis (RA). Overexpression of HDAC6 prompted macrophages to produce TNF-a and IL-6. CKD-506’s inhibitory effect was achieved by blocking NF-ΚB and AP-1 activation (44). (**Figure 2**) HDAC6 inhibition decreased TNF-a and IL-6 production by activated RA PBMCs. CKD-506 also reduced the production of MMP-1, MMP-3, IL-6, and IL-8 by activated FLS. Additionally, CKD-506 inhibited the proliferation of Teffs directly and indirectly by enhancing iTreg function. In AIA rats, oral CKD-506 improved clinical arthritis in a dose-dependent manner (45). A combination of sub-therapeutic CKD-506 and methotrexate demonstrated a synergistic effect (45) (**Table 1**).

M-134, a recently developed HDAC6 inhibitor, was evaluated for its therapeutic potential when combined with tofacitinib in a rat model of rheumatoid arthritis (RA). The single or combined administration of M-134 and tofacitinib was tested in complete Freund’s adjuvant-induced arthritis (AIA) or collagen-induced arthritis (CIA) rodent models. The combination treatment demonstrated strong synergistic effects, as measured by clinical scores and histological changes, without adverse effects such as weight loss in the thymus or increased liver enzymes (ALT and AST). Additionally, it reduced ICAM-1, VCAM-1, and IP-10 expression in the joints. M-134 also enhanced the efficacy of tofacitinib by downregulating various cytokines, including interleukin (IL)-1Β, IL-17, and TNF-a, in the serum of AIA rats (46) (**Figure 2**, **Table 1**).

M808 selectively inhibited HDAC6, reducing the production of MMP-1, MMP-3, IL-6, CCL2, CXCL8, and CXCL10 in RA-FLS cells stimulated by IL-1Β. (**Figure 2**) Increased tubulin acetylation was linked to reduced migration of RA-FLS cells. HDAC6 inhibition led to cytoskeletal reorganization and decreased invadopodia formation in RA-FLS cells activated by IL-1Β. M808 also decreased the expression of ICAM-1 and VCAM-1. In the AIA arthritis model, M808 improved clinical arthritis scores in a dose-dependent manner and was associated with less severe synovial inflammation and joint destruction (47). (**Table 1**) A recent study demonstrated that HDAC6 shRNA-treated mice had significantly lower mean arthritis scores, fewer swollen joints, and reduced paw thickness compared to PBS-treated CIA mice.

Consequently, the inhibition of HDAC6 efficacy in the treatment of RA by significantly reducing inflammatory factors within cells, thereby alleviating symptoms in both collagen-induced arthritis (CIA) and adjuvant-induced arthritis (AIA) mouse models.

#### 3.1.2 Systemic lupus erythematosus

Systemic lupus erythematosus (SLE) is a complex autoimmune disorder affecting multiple organs, marked by the generation of pathogenic antibodies and the formation of immune complexes that may deposit in various tissues (48). Plasma cells (PCs), which are differentiated B cells, play a crucial role in producing antibodies essential for defending against invading pathogens. In lupus, plasma cells (PCs) derived from active B cells generate autoantibodies, including anti-double-stranded DNA (anti-dsDNA) and anti-RNA-binding proteins (49). These autoantibodies bind to self-antigens, forming immune complexes that deposit in blood vessels and renal glomeruli, resulting in vasculitis and nephritis. B cells originate from pluripotent hematopoietic stem cells in the bone marrow (BM). Upon selection of the B cell pathway, B cell development and differentiation proceed through distinct stages, transitioning from proto pre-B to immature B cells (50). Pro-B cells undergo four developmental phases: Phase A (CD24**^−^** BP1**^−^**), Phase B (CD24^+^BP1^−^), Phase C (CD24loBP1^+^), and Phase C′ (CD24^hi^BP1^+^). Pre-B cells progress through three fractions: Fraction D (IgM**^−^** IgD**^−^**), Fraction E (IgM^+^IgD^−^), and Fraction F (IgM**^+^** IgD**^+^**). Fraction D cells undergo recombination of Ig light chains, commence expressing IgM, and differentiate into either fraction E or immature B cells. Fraction E cells then migrate out of the bone marrow and continue their maturation process within the spleen. Upon transitioning from IgM+ immature B cells to IgD expression, they advance to fraction F, also known as mature B cells. Hyperactive B cells significantly contribute to the pathogenesis of Systemic Lupus Erythematosus (SLE) through the stimulation of CD4+ T helper cells, suppression of regulatory T (Treg) cells, release of proinflammatory cytokines, and production of autoantibodies (51). A reduction in Treg cell numbers and functionality has been observed in both mice and humans during active SLE, leading to immune dysregulation and impaired self-tolerance. Furthermore, TH17/Treg imbalance has been associated with the development of inflammatory disorders and renal dysfunction (52). NZB/W mice are generated from the cross of New Zealand Black/BinJ (NZB) and New Zealand White/LacJ (NZW) mice and develop a spontaneous lupus-like disease. Therefore, NZB/W mice serve as an acceptable model of SLE.

Studies have indicated that the selective HDAC6 inhibitor ACY-738, administered to pre-disease lupus-prone NZB/W mice, effectively prevented the onset of lupus nephritis (LN) (53). At 38 weeks of age, NZB/W mice treated with 20 mg/kg ACY-738 exhibited significantly higher numbers of regulatory T cells (Tregs) and lower levels of autoantibody production compared to those receiving vehicle control treatment. Treatment with ACY-738 resulted in an increase in the proportion of B cells at an early developmental stage, while concurrently reducing the proportion of cells within the late pre-B cell fraction (31). Previous studies have demonstrated a correlation between decreased TGF-Β levels in lymphoid tissues and increased autoantibody production, leading to a proinflammatory environment (54). In NZB/W mice, serum TGF-Β levels exhibit age-dependent decline, which is mitigated by ACY-738 treatment in a dose-dependent manner. Mechanistically, the capacity of HDAC6 inhibition to reverse these trends by elevating serum levels of TGF-Β to induce a Treg phenotype, while reducing TGF-Β in the glomeruli of the kidneys, potentially leading to fibrosis (31). (**Figure 2**) Additionally, ACY-738 significantly decreased glucose metabolism and fatty acid oxidation in B cells (**Table 2**).

**Table 2:** HDAC6 inhibitors used as a treatment for rheumatoid arthritis (SLE).

HDAC6 inhibitor(s)	Model	Outcome	Pathogenic mechanism(s)	Reference
ACY-738	NZB/W mice	The deposition of IgG and C3 in glomeruli were significantly decreased	ACY-738 may limit cell metabolism and decrease the spontaneous activation of lupus T and B cells.	(31)
ACY-738	NZB/W mice	ACY-738 reduced serum anti dsDNA and SLE renal pathology	HDAC6 inhibition induces a regulatory T cell (Treg) phenotype by elevating serum TGF-Β levels, while concomitantly reducing TGF-Β expression in renal glomeruli, a process that may promote fibrotic progression.	(54)
CKD-506	NZB/W mice	CKD-506 significantly decreased the incidence of severe proteinuria, blood urea nitrogen, kidney inflammation, and glomerular infiltration of IgG and C3	CKD- 506 signifcantly reduced infammatory cytokines such as IL-10, IL-15, IL-17, TNF-α, and IFN-inducible protein (IP-10) and signifcantly increased TGF-Β in serum.	(43)

In HDAC6 panel assay, CKD-506 inhibited HDAC6 selectively with IC50 value of around 5nM (43). CKD-506 does not inhibit enzyme activity of other HDAC isoforms. CKD-506 significantly improved survival rate and significantly decreased the incidence of severe proteinuria, blood urea nitrogen, kidney inflammation, and glomerular infiltration of IgG and C3. In addition, CKD 506 reduced the proportions of CD138^+^ plasma cells, CD4**^−^** CD8^−^ T cells, and CD25^+^ cells and the Th1/Th2 ratio in the spleen. CKD-506 significantly reduced inflammatory cytokines such as IL-10, IL-15, IL-17, TNF-α, and IFN-inducible protein (IP-10) and significantly increased TGF-Β in serum (43). (**Figure 2**) Thus, CKD-506 decreased the production of various pro-inflammatory cytokines and chemokines in the serum and kidneys, resulting in inhibition of cell migration and suppression of lupus nephritis without adverse effects (**Table 2**).

#### 3.1.3 Psoriasis

**Figure 3:**
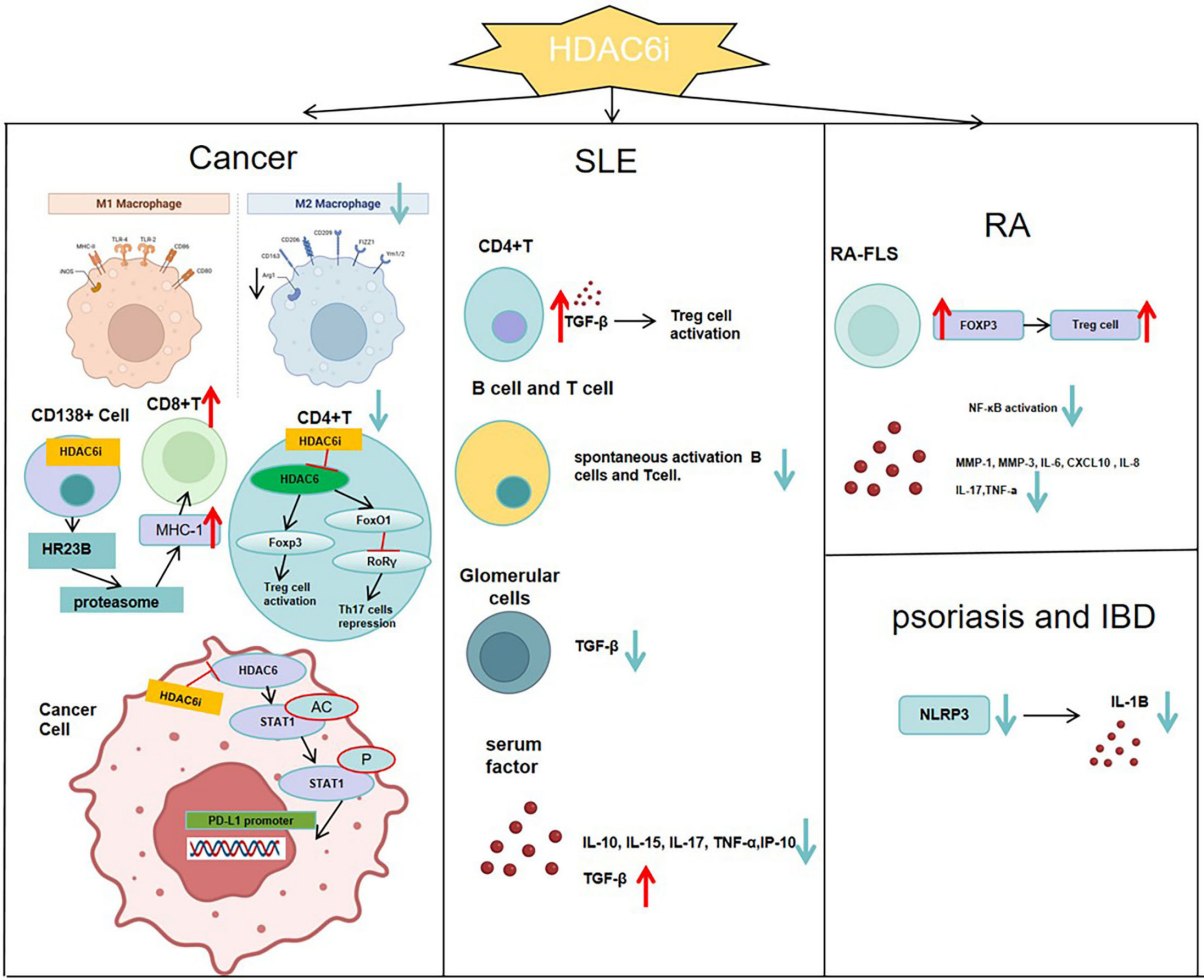
In SLE, inhibiting HDAC6 can promote a Treg phenotype by raising serum TGF-Β levels, while reducing TGF-Β in the glomeruli to slow kidney fibrosis. It also prevents abnormal differentiation of T and B cells and lowers serum levels of cytokines like IL-10, IL-15, IL-17, TNF-α, and IP-10.In RA, inhibiting HDAC6 boosts the suppressive function of Treg cells by raising Foxp3 expression. It also curbs NF-ΚB activation and reduces the production of MMP-1, MMP-3, IL-6, CXCL10, and IL-8 in RA-FLS.In the tumor immune microenvironment, HDAC6 inhibitors enhance the infiltration of CD8+ effector T cells, increase the CD8+/CD4+ cell ratio, inactivate Treg cells, and shift M2-type macrophages toward M1 polarization, thereby reversing the immunosuppressive state of the tumor microenvironment. In CD138^+^ multiple myeloma cells, inhibition of HDAC6 enhances HR23B release, which in turn activates the proteasome, augments MHC class I antigen presentation by tumor cells, and ultimately promotes CD8^+^ T-cell responses.In psoriasis and inflammatory bowel disease, HDAC6 inhibitors prevent the activation of the NLRP3 inflammasome and lower IL-1B levels in the serum.

Psoriasis is a chronic, recurrent, autoimmune, and inflammatory skin disease with a global incidence of approximately 2-4% (55, 56). Clinically, it is characterized by epidermal hyperkeratosis and parakeratosis, vascular changes, and dermal inflammatory infiltration (57). Psoriasis causes symptoms such as itching, silvery scales, and red skin patches. Current treatments primarily include corticosteroids and immunosuppressants (58). These treatments consist of topical agents like ointments with urea, salicylic acid, and glucocorticosteroids, systemic immunomodulators such as methotrexate and cyclosporine, human monoclonal antibodies targeting TNF-α, IL-12, and IL-17, and phototherapy (59, 60). However, these treatments frequently lead to significant side effects, poor patient adherence, immune system disruption, and even liver and kidney toxicity (60). Epigenetic modifications involve heritable changes in gene expression without altering the DNA sequence (61), significantly influencing the interplay between genetic and environmental factors in the development of psoriasis (62). Common epigenetic mechanisms include DNA methylation, post-translational modifications to histone proteins, and noncoding RNA expression. Specifically, histone acetylation and deacetylation, regulated by histone acetyltransferases (HATs) and histone deacetylases (HDACs), are vital components of epigenetic regulation (63). Accumulating evidence suggests that HDAC inhibition may serve as a potential therapeutic option for psoriasis (64). Overexpression of HDAC1 has been observed in psoriatic lesion skin (65), while HDAC3 suppresses the expression of aquaporin-3, a key factor in skin dehydration linked to psoriatic lesions. Additionally, HDAC4, HDAC5, and HDAC6 show altered expression in unaffected skin (66). HDAC1−3 and HDAC6 are upregulated in the macrophage cell line RAW264.7 when induced by lipopolysaccharide (LPS) or imiquimod, effects that can be effectively mitigated by the HDAC inhibitor trichostatin A (TSA) (67). Furthermore, studies indicate that the HDAC inhibitor vorinostat (suberoylanilide hydroxamic acid, SAHA) can inhibit keratinocyte proliferation, offering a promising treatment approach for psoriasis (65, 68). Research on selective inhibitors targeting HDAC6 has been relatively extensive, with traditional compounds still being hydroxyamic acids. Li et al. team previously identified the first hydrazide-based selective HDAC6 inhibitor, 35m, which has advantage in pharmacokinetic properties when compared with traditional hydroxyamic acids. However, subsequent experiments revealed that the insufficient selectivity of 35m toward HDAC1−3 may lead to undesirable side effects at higher dosages (69). Subsequently, their team developed a unique HDAC6 inhibitor through improved design. Ultimately, compound 9m, a highly selective HDAC6 inhibitor, was obtained, demonstrating improved selectivity and lower toxicity compared to compound 35m. 9m inhibits the activation of NLRP3 inflammasome (63), and showed potent oral efficacy significant efficacy in multiple NLRP3-related diseases, including acute peritoneal, inflammatory bowel disease (IBD), and psoriasis (**Figure 3**).

### 3.2 Cancer immunotherapy

Among various HDAC isoforms, HDAC6 has emerged as an excellent target for anti-cancer therapies (70), due to its unique structure and ability to deacetylate numerous non-histone proteins, such as a-tubulin and HSP90 (70), as well as a low likelihood to cause toxicity when inhibited. HDAC6 plays a role in regulating tumor proliferation, invasion, and the tumor immune microenvironment (12, 71). Several HDAC6 inhibitors, such as ACY-1215 and ACY-241 (72, 73), are now in clinical trials for treating various cancers.

#### 3.2.1 Colorectal cancer

Colorectal cancer is one of the most prevalent malignant tumors (74). To date, surgical treatment remains the primary method for managing colorectal cancer in patients without distant metastasis (75). In recent years, targeted therapy and immunotherapy have emerged as primary treatment methods for patients with metastatic colorectal cancer (76). Immune checkpoint inhibitors have become one of the most widely studied immunotherapies and are increasingly used in cancer treatment (77, 78). While some patients experience lasting clinical benefits from immune checkpoint inhibitors, the majority do not respond to this form of immunotherapy. Moreover, some patients develop resistance to immune checkpoint inhibitors even after an initial positive response. Thus, it is crucial for patients to explore effective combined treatment strategies involving these inhibitors (77). We previously reported that the Histone deacetylase (HDAC) family is highly expressed in colorectal cancer specimens and mouse models.

ACY-1215 is an inhibitor of HDAC6, which can inhibit the growth of a variety of tumor. Studies have shown that ACY-1215 combined with anti-PD1 effectively inhibits colorectal tumor growth. Additionally, this combination treatment reduces PD-L1 expression in mouse tumors while upregulating certain biomarkers associated with T cell activation (79). Human colorectal cancer cell lines include HCT116 and SW480. In a co-culture system of T cells and colorectal cancer cells, ACY-1215 enhanced the ability of T cells to kill HCT116 cells. Mechanistically, HDAC6 increased the acetylation of STAT1 and inhibited its phosphorylation, thereby preventing STAT1 from entering the nucleus and activating PD-L1 transcription (79). (**Figure 2**) Thus, HDAC6 inhibitors may be of great significance in colorectal cancer immunotherapy (**Table 3**).

**Table 3:** HDAC6 inhibitors used as a treatment for cancer.

HDAC6 inhibitor(s)	Model	Outcome	Pathogenic mechanism(s)	Reference
ACY-1215	HCT116 and SW480 cell line	Efectively inhibited the colorectal tumor growth.	HDAC6 enhanced the acetylation of STAT1 and inhibited the phosphorylation of STAT1, thus preventing STAT1 from entering the nucleus to activate PD-L1 transcription.	(79)
AVS100		Prevent the growth of melanoma	It can boost T cell infiltration, decrease M2 macrophage polarization in the tumor microenvironment, and significantly curb tumor growth.	(26)
HDAC6 -KO	A549 and H1299 cell lines	Inhibit the growth of lung cancer cells	In terms of treatment selection, HDAC6 inhibitors may enhance the efficacy of immunotherapy by modulating the tumor microenvironment (e.g., promoting M1 macrophage polarization and inhibiting Treg infiltration).	(83)
Tubastatin-A or ACY-738	CD138^+^ cells	Increase the expression level of MHC class I antigens and augment CD8^+^ T-cell responses.	HDAC6 inhibitors act on CD138^+^ cells, promote the release of HR23B to enhance proteasomal activity, thereby increasing the expression level of MHC class I antigens and augmenting CD8^+^ T-cell responses.	(85)

#### 3.2.2 Melanoma

HDAC6 inhibitors have shown significant immune-modulating effects in melanoma treatment. Specifically, HDAC6 exerts its immune-regulatory function by modulating the expression of immune-suppressive molecules IL-10 and PD-L1. The regulation of these molecules enhances the effectiveness of anti-PD-1 immune checkpoint blockade therapy, thereby boosting anti-tumor immune responses. In actual research, using selective HDAC6 inhibitors in combination with anti-PD-1 immune checkpoint blockade therapy significantly reduces tumor growth. For example, AVS100, as a selective HDAC6 inhibitor, enhances T-cell infiltration and reduces the polarization of M2 macrophages in the tumor microenvironment when used in combination with anti-PD-1. This significantly inhibits tumor growth and results in higher survival rates compared to single treatments (26). Moreover, HDAC6 inhibitors also influence PD-L1 expression by affecting other molecules such as MIIP (a potential melanoma immune modulator) (80), thereby enhancing the efficacy of immune checkpoint inhibitors. These findings highlight the potential of combining HDAC6 inhibitors with anti-PD-1 immune checkpoint blockade therapy, offering new approaches for melanoma treatment. In summary, HDAC6 inhibitors have a positive biological basis and clinical application potential for melanoma treatment through regulating the expression of key immune molecules and enhancing immune responses. Additionally, ACY-1215 or ACY-241, was found to remarkably reduce Foxp3 expression, thereby inhibiting the immune suppressive function of Treg cells in melanoma patients (81) (**Table 3**).

#### 3.2.3 Lung cancer

Lung cancer is the second most prevalent cancer globally, causing about 1.8 million deaths annually (82). Lung adenocarcinoma (LUAD) is the most common histological subtype, making up nearly 40% of all lung cancer cases. Research has shown that high HDAC6 expression in LUAD is linked to an immunosuppressive tumor microenvironment, marked by low immune scores, reduced infiltration of B cells and CD8+ T cells, and increased infiltration of suppressive immune cells such as CAFs and MDSCs. A recent study found that inhibition or ko of HDAC6 inhibited tumor growth, suppresses PI3K/AKT/mTOR signaling and epithelial-mesenchymal transition (EMT), and enhances apoptosis and M1 macrophage recruitment (83). Therefore, HDAC6 is a significant prognostic marker and therapeutic target in LUAD (**Table 3**).

#### 3.2.4 Multiple myeloma

In the tumor microenvironment, low-dose HDAC inhibitors can reduce the number of regulatory T cells (Treg), abrogate the inhibitory effect of Treg on CD8^+^ T cells, and enable CD8^+^ T cells to more effectively recognize and kill tumor cells expressing MHC class I antigens. Consequently, this promotes the expression and presentation of MHC class I antigens to a certain extent (84). A study indicated that HDAC6 inhibitors enhance proteasome activity, thereby amplifying and expanding MHC-I antigen presentation on myeloma cells and promoting CD8+ T cell responses. Treatment of patient CD138+ cells with HDAC6 inhibitors significantly enhanced the antimyeloma activity of autologous CD8+ T cells. Pharmacological blockade and genetic ablation of the HDAC6 ubiquitin-binding domain released HR23B, which transports ubiquitinylated cargo to proteasomes. Silencing HDAC6 or HR23B in multiple myeloma cells nullified the impact of HDAC6 inhibitors on proteasomes, antigen presentation, and T-cell cytotoxicity. (85). Thus, in multiple myeloma, HDAC6 inhibitors act on CD138^+^ cells, promote the release of HR23B to enhance proteasomal activity, thereby increasing the expression level of MHC class I antigens and augmenting CD8^+^ T-cell responses (**Figure 3**).

### 3.3 Neuroinflammation

#### 3.3.1 Alzheimer’s disease

Alzheimer’s disease (AD) is the most prevalent neurodegenerative disorder leading to dementia. The primary pathological characteristics of AD include intracellular accumulation of pathological tau proteins forming neurofibrillary tangles (NFTs) and extracellular deposition of beta-amyloid (AΒ) as senile plaques. In physiological conditions, tau regulates microtubule dynamics; however, in a pathological state, tau becomes highly phosphorylated and aggregates to form NFTs in AD. This hyperphosphorylated and aggregated tau results in synaptic dysfunction, mitochondrial damage, and ultimately neuronal cell death (86–88). In recent years, significant interest has emerged regarding the relationship between HDAC6 and Alzheimer’s Disease (AD). Numerous studies have identified elevated levels of HDAC6 in the brains of AD patients (89), leading to decreased acetylated α-tubulin levels and subsequent neuronal dysfunction (89).

The therapeutic potential of HDAC6 inhibitors (HDAC6is) is garnering increased attention following pharmacological treatments of certain selective HDAC6is in Alzheimer’s disease (AD) models, which have demonstrated reductions in tau levels, improvements in axonal transport, restoration of learning and memory, and anti-inflammatory activity (90, 91). Currently, the pharmacophore of HDAC6 inhibitors employed in clinical settings for the treatment of AD typically comprises three distinct domains:zinc-binding group, surface-recognizing capping group, and a linker between them (92). In the latest study, researchers developed a potent HDAC6 inhibitor called PB118, which features strong binding affinity, selectivity, and good brain penetration based on the characteristics of these inhibitors. The regulation of brain function relies on the phagocytic activity of glial cells. In the early stages of Alzheimer’s disease (AD), this process helps protect against AΒ accumulation (86). However, in later stages, impaired phagocytic function in glial cells can lead to worse disease outcomes. To evaluate the impact of PB118 on microglial phagocytic regulation, researchers treated mouse microglial cells BV2 with PB118 in the presence or absence of AΒ and conducted a series of experiments. Compared to the control group, PB118 increased microglial phagocytosis of AΒ. Using PB118 in BV2 cells significantly elevated acetylated α-tubulin, stabilizing the intracellular microtubule network. The results showed that PB118 significantly reduced interleukin-6 (IL-6), CXCL1, and IL-12p-70. PB118 is non-toxic to human neural cells, and high concentrations of PB118 can significantly lower p-tau and total tau levels in the system (93). In summary, these data indicate that PB118 alleviates AD symptoms through multiple mechanisms, making it a highly promising clinical molecule for AD (**Table 4**).

**Table 4:** HDAC6 inhibitors used as a treatment for neuritis.

Disease	HDAC6 inhibitor(s)	Model	Outcome	Pathogenic mechanism(s)	Reference
Alzheimer's disease (AD)	PB118	BV2 cells	PB118 alleviates AD symptoms	PB118 significantly reduced IL-6, CXCL1, and IL-12p-70.	(93)
Neuropathic pain (NP)	ACY-1215	spinal nerve ligation (SNL) was used in rats.	Mechanical allodynia, cognitive impairment, and depressive-like behavior resulting from SNL were reduced	Administration of ACY-1215 effectively mitigated SNL-induced neuroinflammatory responses, including microgliosis and the elevation of pro-inflammatory cytokines IL-1Β and TNFα, within the ipsilateral spinal dorsal horn (iSDH), hippocampus (HPC), and prefrontal cortex (PFC).	(103)
Chemotherapy-induced peripheral neuropathy	ACY-1083	The mice were administered two cycles of cisplatin	ACY-1083 prevented cisplatin-induced mechanical and also completely reversed allodynia, spontaneous pain, and numbness.	Mechanistically, inhibition of HDAC6 mitigates CIPN by enhancing mitochondrial health, elevating α-tubulin acetylation, and facilitating the transport of functional mitochondria to the peripheral terminals of sensory neurons.	(107)
Alzheimer's disease (AD)	CKD-504	Alzheimer's disease-like pathology ADLP^APT^ mice	CKD-504 significantly reduced pathological tau and rescued synaptic pathologies and cognitive impairment in ADLP^APT^ mice	CKD-504 also regulated acetylation of tau and the chaperone proteins such as Hsc70 and Hsp70, followed by ubiquitination and proteasomal degradation of tau.	(94, 95)

CKD-504 is a highly blood-brain barrier (BBB)-penetrating HDAC6 inhibitor (94). The study found that CKD-504 reduced tau in AD patient-induced pluripotent stem cells (iPSCs)-derived brain organoids. In addition, CKD-504 significantly reduced pathological tau and rescued synaptic pathologies and cognitive impairment in ADLP^APT^ (Alzheimer’s disease-like pathology^APP & Tau^) mice (95). CKD-504 also regulated acetylation of tau and the chaperone proteins such as Hsc70 and Hsp70, enhancing the interactions among these proteins as well as the recruitment of the novel tau E3 ligases (UBE2O and RNF14), followed by ubiquitination and proteasomal degradation of tau (94). Moreover, they revealed that acetylation on K274, K290, K321, and K353 of tau is involved in proteasomal degradation of tau by CKD-504 (94).

#### 3.3.2 Neuropathic pain

Neuropathic pain (NP) is defined as ‘pain resulting from a lesion or disease of the somatosensory nervous system,’ with a prevalence ranging between 6% and 8% in the general population (96, 97). Over 60% of patients suffering from neuropathic pain also experience depression, a condition marked by prolonged and persistent depressive symptoms coupled with diminished motivational drive (98). During inflammation, the expression of histone deacetylases (HDACs) increases (99, 100), leading to deacetylation of NF-kB and promoting the production of inflammatory factors (101). Inhibition of HDAC6 regulates the acetylation of lysine of NF-ΚB in the cytoplasm (102), and prevents its nuclear translocation. This unique regulatory pathway leads to the suppression of NF-ΚB transcriptional activity, culminating in the manifestation of anti-inflammatory and analgesic properties (33, 103, 104).

In this study, spinal nerve ligation (SNL) was used as a neuropathic pain model in rats. Mechanical allodynia, cognitive impairment, and depressive-like behavior resulting from SNL were reduced by continuous intraperitoneal injection of ACY-1215. Moreover, administration of ACY-1215 effectively mitigated SNL-induced neuroinflammatory responses, including microgliosis and the elevation of pro-inflammatory cytokines IL-1Β and TNFα, within the ipsilateral spinal dorsal horn (iSDH), hippocampus (HPC), and prefrontal cortex (PFC) (103). Mechanistically, the MyD88-dependent pro-inflammatory pathways, specifically MyD88/NF-ΚB and MyD88/ERK, were activated in the iSDH following SNL and were subsequently inhibited by ACY-1215 (103) (**Table 4**).

#### 3.3.3 Chemotherapy-induced peripheral neuropathy

Chemotherapy-induced peripheral neuropathy(CIPN)represents one of the most prevalent dose-limiting side effects associated with cancer therapy (105). At present, no treatment has been approved by the Food and Drug Administration for this condition (106). Karen et al. used a novel HDAC6 inhibitor ACY-1083, which shows 260-fold selectivity towards HDAC6 vs other HDACs. Karen et al. discovered ACY-1083 prevented cisplatin-induced mechanical allodynia, and also completely reversed allodynia, spontaneous pain, and numbness. The mice were administered two cycles of cisplatin, resulting in a cumulative dose of 23 mg/kg (107). Three days following the last cisplatin administration, when mechanical allodynia was already evident, the mice received seven consecutive daily doses of ACY-1083 at either 3 mg/kg or 10 mg/kg, or a vehicle control. Treatment with 10 mg/kg of ACY-1083 successfully alleviated cisplatin-induced mechanical allodynia, whereas the 3 mg/kg dose did not (107). They also tested the HDAC6 inhibitor, ACY-1215 (Ricolinostat). Mice received two rounds of cisplatin treatment, followed by oral doses of ACY-1215 at 30 mg/kg daily for two weeks, starting three days after the last cisplatin dose. The results showed that daily administration ACY-1215 effectively reversed cisplatin-induced mechanical allodynia. The beneficial effects of ACY-1215 were still evident one week after treatment completion. Mechanistically, inhibition of HDAC6 mitigates CIPN by enhancing mitochondrial health, elevating α-tubulin acetylation, and facilitating the transport of functional mitochondria to the peripheral terminals of sensory neurons (107). Ma et al. demonstrated that in both male and female mice, pharmacological inhibition of HDAC6 using ACY-1215 or global deletion of HDAC6 is sufficient to prevent cisplatin-induced mechanical allodynia, loss of intraepidermal nerve fibers (IENFs), as well as mitochondrial bioenergetic deficits in dorsal root ganglion neurons and peripheral nerves. Furthermore, deletion of HDAC6 specifically in sensory neurons protects against cisplatin-induced loss of IENFs and the reduction in mitochondrial bioenergetics and content in peripheral nerves. These findings clarify the specific functions of HDAC6 in the CIPN model and provide a mechanistic basis for the development of HDAC6-targeted strategies for the prevention and treatment of CIPN (108) (**Table 4**).

## 4 The potential applications of HDAC6 inhibitors in clinical settings

### 4.1 HDAC6 inhibitors in clinical trials

Currently, various inhibitors are utilized in clinical practice. ACY-1215 also had a phase I clinical trial (NCT02632071) in which it was coupled with nab-paclitaxel to treat metastatic breast cancer (109–111). ACY-241, also named Citarinostat, is a bioavailable second generation selective HDAC6 inhibitor. ACY-241 has successfully completed a Phase I clinical trial (NCT02551185) for advanced solid tumors. Furthermore, a Phase I clinical trial (NCT02635061) has also been completed to evaluate the potential of combining ACY-241 with the PD-1 inhibitor Nivolumab in patients with non-small cell lung cancer (109, 112, 113). This study aims to investigate whether HDAC6 inhibition can enhance the efficacy of immunotherapy and standard treatments. Currently, KA2507, another HDAC6 inhibitor developed by Karus Therapeutics Limited, has completed a phase I clinical trial (NCT03008018) in solid tumors (adult) and a phase II clinical trial (NCT04186156) in biliary tract cancer has been retracted (70, 109).

### 4.2 Challenges and perspectives

In cancer therapy, reversible HDAC6 inhibitors, including ACY-1215 and ACY-241, have progressed to clinical research. Nonetheless, the selectivity of HDAC6 in these compounds remains relatively low, approximately tenfold compared to HDAC1/2/3 (114), and still fails to entirely mitigate the adverse effects associated with other HDAC subtypes. Furthermore, they encounter the prevalent challenge of poor metabolic stability characteristic of oxime derivatives, necessitating high dosages and repeated administrations to sustain adequate blood concentrations and therapeutic effective (115). The development of irreversible inhibitors of HDAC6 that exhibit high subtype selectivity and prolonged efficacy on the target represents an effective strategy to address the aforementioned limitations of current inhibitors. Besides improving the subtype selectivity of the inhibitor, a combination strategy might be better. Multiple studies have demonstrated that dual inhibitors significantly enhance therapeutic efficacy across various diseases. In the tumor immune microenvironment, AVS100 enhance the infiltration of CD8+ effector T cells, inactivate Treg cells, and shift M2-type macrophages toward M1 polarization, thereby reversing the immunosuppressive state of the tumor microenvironment. Specifically, the HDAC6 inhibitor AVS100 in combination with anti-PD-1 therapy synergistically potentiates T-cell immune responses, overcoming tumor immune tolerance (26). A dual sEH/HDAC6 inhibitor reported in JMC alleviates neuropathic pain, while HDAC6/HSP90 dual inhibitors reverse immunosuppressive tumor microenvironments (104). Additionally, JAK2/HDAC6 dual inhibitors effectively ameliorate pathological symptoms in psoriasis mouse models, highlighting the translational potential of multi-targeted therapeutic strategies (69).

## 5 Conclusion

In contrast to other histone deacetylases (HDACs), class IIb deacetylases comprise HDAC6 and HDAC10. Similar to HDAC6, HDAC10 features a dual HDAC domain structure. However, only one of these domains, the polyamine deacetylase (PADC), exhibits deacetylase activity. The second domain (ΨDAC) does not contain substrate and Zn²^+^ binding sites or catalytic residues, rendering it non-catalytic; nonetheless, it contributes to the structural stability of PADC (116). HDAC6 possesses two functional catalytic domains and a ubiquitin-binding region characterized by a zinc finger structure (ZnF-UBP). This unique configuration enables HDAC6 to engage with diverse proteins. Consequently, HDAC6 plays a pivotal role in regulating immune cell function, inflammatory responses, and protein homeostasis via multiple pathways. Accumulating evidence has highlighted that small-molecule inhibitors targeting histone deacetylase 6 (HDAC6) hold therapeutic potential for autoimmune diseases, tumor immune evasion, and neuroimmune disorders. In melanoma, HDAC6 inhibitors substantially enhance the infiltration of CD8^+^ effector T cells, thereby increasing the CD8^+^/CD4^+^ cell ratio. Furthermore, HDAC6 inhibitors have the capacity to induce regulatory T cells (Tregs) and M2-type macrophages to adopt anti-tumor phenotypes. This process leads to the deactivation of Treg cells and facilitates the polarization of M2-type macrophages towards M1-type, thereby fundamentally reversing the immunosuppressive condition within the tumor microenvironment. The N-terminus of HR23B harbors a ubiquitin-like domain (UBL) that interacts with the 26S proteasome, facilitating the translocation of ubiquitinated proteins to the proteasome and thereby contributing to ubiquitin-mediated protein degradation. In CD138^+^ multiple myeloma cells, inhibition of HDAC6 enhances HR23B release, which in turn activates the proteasome, augments MHC class I antigen presentation by tumor cells, and ultimately promotes CD8^+^ T-cell responses. In colon cancer, the HDAC6 inhibitor ACY-1215 not only enhances T-cell cytotoxic activity but also downregulates PD-L1 expression on the surface of tumor cells, alleviating immune suppression. In lung cancer, HDAC6 inhibitors strengthen the local pro-inflammatory immune microenvironment by promoting the recruitment of M1-type macrophages. Taken together, HDAC6 exerts a significant regulatory effect on cancer-related immune disorders.

During the neuropathological process of neuroinflammation, HDAC6 functions as a deacetylase for alpha-tubulin. Inhibition of HDAC6 can increase the acetylation level of α-tubulin by blocking the deacetylation process of tubulin. Maintaining the acetylated state of microtubules enhances the stability of the cytoskeleton, thereby significantly promoting axonal growth and synaptic plasticity of neurons, and providing the necessary structural support for neural repair and regeneration. However, merely detecting the acetylation level at lysine 40 (K40) of tubulin is insufficient to fully reflect its ultimate biological effects. In fact, the synergistic interaction between acetylation modifications (including those on other lysine residues) and other post-translational modifications is likely the key determinant of its function. Furthermore, inhibition of HDAC6 mitigates CIPN by enhancing mitochondrial health, elevating α-tubulin acetylation, and facilitating the transport of functional mitochondria to the peripheral terminals of sensory neuron. Additionally, HDAC6 inhibitors can effectively suppress the activation and proliferation of microglia, reduce the release of inflammatory factors and cytotoxic substances, and mitigate neural damage from an immunoregulatory standpoint, thereby offering a multidimensional intervention strategy for the pathological improvement of neuroinflammation.

In RA and SLE, HDAC6 inhibitors promote the induction of regulatory T cell (Treg) phenotypes and concurrently reduce inflammatory factors within the serum, thereby mitigating disease progression. The ubiquitin-binding domain of HDAC6 is critical for the trafficking and activation of the NLRP3 inflammasome. Specifically, the zinc finger domain of HDAC6 interacts with ubiquitinated NLRP3, recruiting it to microtubules and facilitating its trafficking. In atherosclerosis, the HDAC6 inhibitor Tubastatin A reduces macrophage foam cell formation, thereby inhibiting plaque progression. In psoriasis, blocking the assembly of the NLRP3 inflammasome complex effectively suppresses the secretion of inflammatory cytokines.

This review offers a structural characteristics and biological functions of HDAC6, along with the therapeutic mechanisms of selective inhibitors in treating immune disorders. Moreover, this review seeks to provide a theoretical framework and research direction for the development of novel HDAC6 inhibitors, thereby advancing precise treatments for immune disorders.
